# *Rubus apetalus* (Rosaceae) improves spermatozoa characteristics, antioxidant enzymes and fertility potential in unilateral cryptorchid rats

**DOI:** 10.1186/s12610-020-00107-3

**Published:** 2020-07-09

**Authors:** Désiré Alumeti Munyali, Aimé Césaire Tetsatsi Momo, Georges Romeo Bonsou Fozin, Patrick Brice Deeh Defo, Yannick Petnga Tchatat, Boris Lieunang, Pierre Watcho

**Affiliations:** 1 Research Unit of Animal Physiology and Phytopharmacology (URPAP), Faculty of Science, University of Dschang, Dschang, Box 67, Dschang, Cameroon; 2 School of Medicine and Community Health, Université Evangelique en Afrique, Bukavu, Democratic Republic of Congo

**Keywords:** Cryptorchidism, *Rubus apetalus*, Oxidative stress, Fertility, Rat, Cryptorchidie, *Rubus apetalus*, Stress oxydatif, Fertilité, Rat

## Abstract

**Background:**

Cryptorchidism (CPT) is an important cause of male infertility. *Rubus apetalus* is a medicinal plant with a powerful antioxidant potential. We investigated the effects of aqueous and methanolic extracts of *R. apetalus* on spermatozoa parameters, antioxidant enzymes and fertility potential of rats with experimental unilateral CPT.

**Method:**

Normal (*n* = 15), sham-operated (*n* = 15) and cryptorchid rats (*n* = 80; distributed into 16 groups of 5 rats/group) were treated for 2, 4 or 8 weeks with either distilled water (10 ml/kg/day), vitamin E (75 mg/kg/day), aqueous or methanolic extract of *R. apetalus* (12 and 60 mg/kg). Sex organ weights, spermatozoa parameters, testicular proteins, sex hormones, fertility potential, morphometric characteristics of testis and oxidative stress markers were measured.

**Results:**

CPT significantly (*p* < 0.05–0.001) decreased testicular and epididymal weights, spermatozoa density, spermatozoa motility, spermatozoa normality, testicular proteins, LH, FSH and testosterone concentrations. In cryptorchid rats, peri-vascular fibrosis significantly increased (*p* < 0.001), while diameter of the seminiferous tube, germ cell thickness, gestation index and fertility index decreased when compared to control. Additionally, CPT induced oxidative stress by increasing lipid peroxidation and by reducing superoxide dismutase and catalase activities. These alterations were corrected by *R. apetalus.* For instance, a significantly increase (*p* < 0.05–0.001) in spermatozoa motility, normality, viability and density after 2, 4 and 8 weeks of treatment was noticed. *R. apetalus* also increased (*p* < 0.05–0.001) testicular proteins, gestation index (90–100%) and fertility index (90–100%), compared to the untreated cryptorchid rats.

**Conclusion:**

*R. apetalus* boosts fertility potential in cryptorchid rats and could be considered as a promising alternative agent for the management of infertility associated with CPT.

## Résumé

**Contexte et justificatif:**

La cryptorchidie (CPT) est. une importante cause de l’infertilité masculine dans le monde. *Rubus apetalus* est. une plante médicinale au potentiel antioxydant avéré. L’objectif de ce travail était d’évaluer les effets des extraits aqueux et méthanolique de *R. apetalus* sur les paramètres spermatiques, les enzymes antioxydantes et le potentiel de fertilité des rats à CPT unilatérale.

**Méthode:**

Les rats normaux (*n* = 15), simulés (*n* = 15) et cryptorchides (*n* = 80; répartis en 16 groupes de 5 rats/groupe) ont été traités pendant 2, 4 ou 8 semaines avec de l’eau distillée (10 ml/kg/jour), la vitamine E (75 mg/kg/jour) ou l’extrait aqueux ou méthanolique de *R. apetalus* (12 et 60 mg/kg). La masse des organes sexuels, les caractéristiques spermatiques, les marqueurs du stress oxydatif, les hormones sexuelles, l’histomorphométrie testiculaire et la fertilité ont été mesurés au terme des traitements.

**Résultats:**

La CPT a significativement diminué (*p* < 0,05-0,001) la masse testiculaire et épididymaire du côté cryptorchide. Le même constat était fait par rapport à la densité, à la motilité, à la normalité spermatique ainsi que les protéines testiculaires et les hormones sexuelles (LH, FSH et testostérone plasmatiques). Chez les rats cryptorchides, la fibrose péri-vasculaire a augmenté de manière significative (*p* < 0,001) tandis que le diamètre des tubes séminifères, l’épaisseur des cellules germinales, l’indice de gestation et l’indice de fertilité ont diminué par rapport au témoin. De plus, le CPT a induit un stress oxydatif caractérisé par la peroxydation lipidique et la réduction de l’activité des enzymes antioxydantes (SOD et catalase). Ces altérations ont été corrigées par *R. apetalus*. Une augmentation significative (*p* < 0,05-0,001) des paramètres spermatiques, des protéines testiculaires et des hormones sexuelles a été notée après 2, 4 et 8 semaines de traitement aux extraits de plante. *R. apetalus* a par ailleurs normalisé l’activité des enzymes antioxydantes et augmenté l’indice de gestation (90–100%) et l’indice de fertilité (90–100%) par rapport aux rats cryptorchides non traités.

**Conclusion:**

*R. apetalus* augmente le potentiel de fertilité chez les rats cryptorchides et pourrait être une alternative prometteuse pour la prise en charge de l’infertilité masculine due à la cryptorchidie.

## Introduction

Cryptorchidism (CPT) is a boy-congenital malformation characterized by the absence of one (unilateral CPT) or both (bilateral CPT) testes in the scrotum [1, 2]. Evidence from literature indicates that unilateral CPT is more frequent than bilateral CPT [3, 4]. Among unilateral CPT cases, right unilateral CPT is the more expressed [5–7]. CPT impairs testicular function (mainly due to high temperature) and contributes to male infertility [8]. Although the etiology of this disease is unclear, genetic factors, environmental pollution, nutrition and lifestyle are the main causes of CPT. In the testes of patients with CPT, the integrity of the blood-testis-barrier is compromised due to high temperature [9]. Indeed, high scrotal temperature induces lipid peroxidation, overproduction of reactive oxygen species (ROS) and decreases antioxidant enzymes (catalase, superoxide dismutase, glutathione peroxidase), leading to germ cells death and infertility [10, 11]. CPT-induced infertility is characterized by a decrease in spermatozoa motility and azoospermia due to the destruction of germ cells and apoptosis [12].

Treatment of CPT is essentially surgical [7] and based on the repositioning of the cryptorchid testis in the normal position (inside the scrotum) in order to protect it against high temperature and oxidative stress [13–15]. Antioxidant compounds such as vitamin E are also used as adjuvant treatment to control apoptosis of the germ cells by decreasing oxidative stress which is the main cause of the detrimental effects of CPT [16]. These treatment options are expensive and not accessible to low income population. The effectiveness of some medicinal plants in the management of CPT has been reported. For example, *Moringa oleifera* [17, 18] improves spermatozoa parameters in cryptorchid rats through its powerful antioxidant potential; *Cuscuta chinensis* decreases the germ cell apoptosis in unilateral cryptorchid rats by improving the antioxidant enzymes and regulating the expression of Bax, Bcl-2 and, cleaved caspase 3 [19].

*Rubus apetalus,* one of such plants, is an annual scrambling shrub from the *Rosaceae* family. This tropical plant has a cluster of stems from a woody rootstock and can grow to around 150 cm tall [20]. Its fruits are used as a local food, though the plant is sometimes cultivated in gardens [21, 22]. A decoction of the ripe fruit is mixed with sugar and used to treat anemia while an infusion of the leaves is used for the management of diabetes mellitus [23]. *R. apetalus* is also used as fertility booster [24], anti-venom [25], anti-fungi [20]*,* and anti-bacteria [26]. Moreover, the in vitro [27, 28] and in vivo antioxidant potentials of *R. Apetalus* have been reported and could be justified by its contents in bioactive compounds such as saponins, alkaloids, flavonoids, tannins, triterpenoids and vitamins [23]. Since oxidative stress is considered as the main pathway in the harmful effects of CPT, we hypothesized that the powerful antioxidant potentials of *R. Apetalus* may prevent testicular damages and consequently improve fertility. The present study was therefore undertaken to investigate the pharmacological effects of the aqueous and methanolic extracts of *R. apetalus* on some reproductive (sex organ weights, spermatozoa characteristics, testicular proteins, sex hormones, fertility potential and morphometric characteristics of testis) and metabolic (oxidative stress-related biochemical markers) parameters in rats with right unilateral CPT.

## Materials and methods

### Plant collection

*Rubus apetalus* (Rosaceae) leaves were harvested in February 2018 in Katana, East Region of Democratic Republic of Congo. The plant was identified by an ethnobotanist (Mr. Balagizi Karhagomba) and authenticated at the Natural Science Research Center (CRSN) under the voucher number 181. The leaves were shade-dried and transformed into powder with an electric grinder and the powder obtained was used for preparation of extracts.

### Preparation of extracts

Aqueous extract was obtained by infusing *R. apetalus* powder (5 g) in distilled water (500 ml). After filtration, the filtrate was oven-dried at 40 °C and 1.054 g of residue was obtained (extraction yield: 21.08%).

For the methanolic extract, 5 g of *R. apetalus* powder were macerated in 500 ml of methanol (95%) for 72 h. After filtration, the filtrate was evaporated under reduced pressure using a rotative evaporator and 0.516 g of residue (methanolic extract) was obtained, giving an extraction yield of 10.32%.

The working solutions of aqueous and methanolic extracts of *R. apetalus* were prepared in distilled water and administered at 12 mg/kg/day and 60 mg/kg/day. Doses of plant extracts were chosen from our pilot studies (unpublished data) while that of vitamin E was based from our previous work [29].

### Animals

Young male Wistar rats (8 weeks old; 120–140 g body weight) were obtained from the animal house of the Faculty of Science, University of Dschang, Cameroon. They were maintained in a standard environment (22–25 °C; 12 h of light and 12 h of dark cycle) and had food and water ad libitum. The project was presented and validated by the scientific committee of the Department of Animal Biology, University of Dschang, which follows the internationally accepted standard ethical guidelines for laboratory animal use and care as described in the European Economic Community guidelines; EEC. 2010 Council Directive 2010/63/EU of 22 November 2010 [30].

### Induction of cryptorchidism

Right unilateral CPT is the most common form of CPT [1, 7]. Right unilateral CPT was induced using the method of Toshihiko et al. [31] with minor modifications. Briefly, the rats were anesthetized with an intra-peritoneal administration of diazepam (10 mg/kg), followed 10 min later by ketamine (50 mg/kg). Once the anesthesia was effective, the anterior part of the abdomen was shaved from the right flank to the right ilia fossa in an aseptic condition. An oblique incision (about 2 cm) was made, followed by a section of the abdominal muscles, an opening of the parietal peritoneum and, the identification of *gubernaculum testis* and right inguinal canal. In order to free the bursa, the *gubernaculum testis* was cut, the right testis was then pulled into the abdomen and the inguinal canal was sutured with cotton thread which prevented further descent of the testis into the scrotum. The surgical procedure ended with repositioning the testis in the abdomen, suturing the peritoneum and muscle before suturing the skin with a cotton thread. After surgery, rats were intramuscularly administered with antibiotic to prevent infections. Cryptorchid rats were used for further experiments 3 days after induction of cryptorchidism [17, 32, 33].

### Experimental protocol

**Fig. 1:**
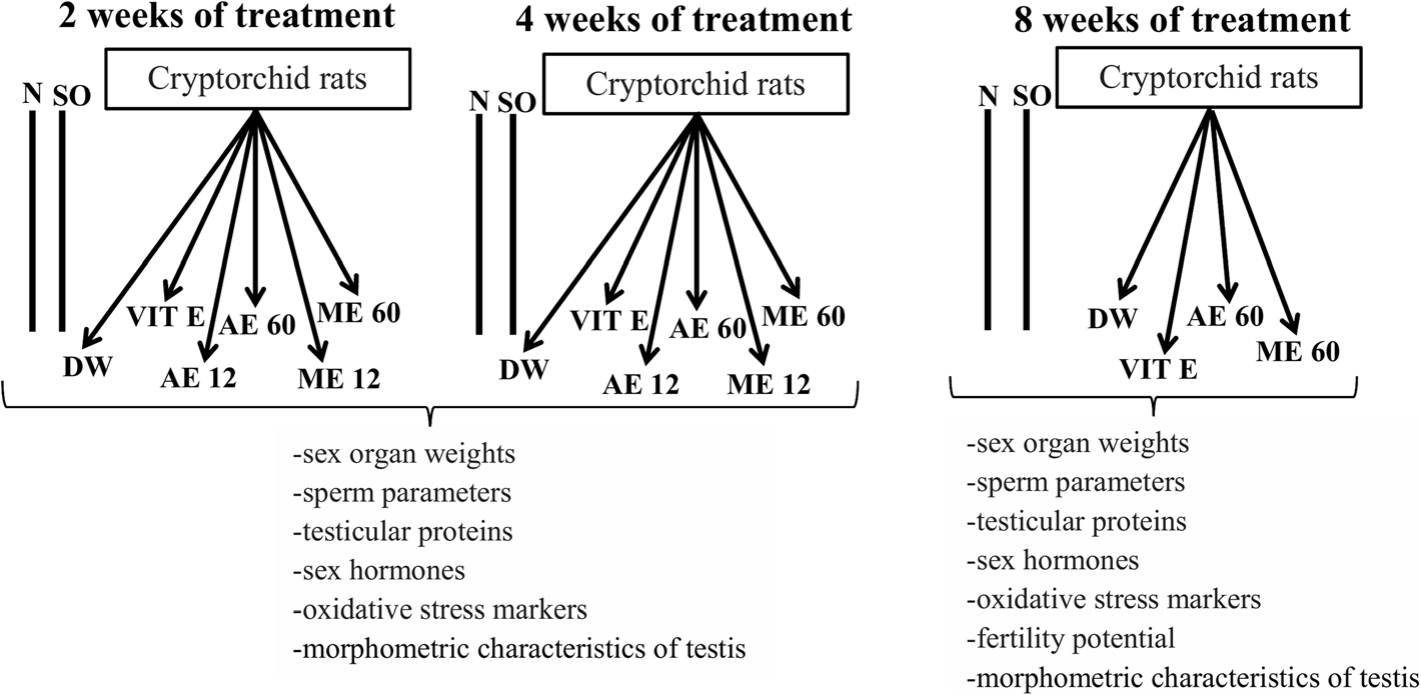
Experimental protocol of study. Number of rats per group = 5. Abbreviations: N: normal; SO: sham operated; DW: distilled water; Vit E: vitamin E; AE12 / AE60: aqueous extract administered at 12 mg/kg or 60 mg/kg; ME12 / ME60: methanolic extract administered at 12 mg/kg or 60 mg/kg

One hundred ten rats (15 normal, 15 sham operated and 80 cryptorchid rats) were randomly distributed into 22 groups of 5 animals each and orally treated for 2, 4 or 8 weeks with either distilled water (10 ml/kg/day), vitamin E (75 mg/kg/day), aqueous or methanolic extract of *R. apetalus* (12 and 60 mg/kg). Only the most effective dose of plant extract (60 mg/kg) was administered to rats for 8 weeks, the period required for a complete spermatogenic cycle in rats [34] (Fig. 1).

At the end of each treatment period (2, 4 and 8 weeks), testicular and epididymal weights, spermatozoa characteristics, oxidative stress markers, testicular proteins, sex hormones and morphometric characteristics of testis were measured. Moreover, fertility potential was evaluated at the end of the last treatment period (week 8). Indeed, each male was mated with two females of proven fertility. Vaginal smear was daily examined under light microscope (OLYMPUS, X40). The presence of spermatozoa in the vaginal smear was taken as criteria of successful insemination and considered as day 1 of pregnancy. Fertility potential (gestation index, fertility index and litter size) was recorded as described previously [35, 36].

### Tissue preparation and sample analysis

At the end of each treatment period, rats were sacrificed under diazepam/ketamine anaesthesia and testes and epididymis were removed. Relative sex organ weights were calculated using the following formula: Relative sex organ weight = (absolute sexual organ weight/body weight) × 100. Half of each epididymis was used for spermatozoa characteristics and the rest for measurement of oxidative stress markers including malondialdehyde (MDA), superoxide dismutase (SOD) and catalase (CAT) and total protein levels. Abdominal artery blood was collected into heparinized tubes and centrifuged (3000 g for 10 min). The plasma obtained was used to quantify sex hormones including luteinizing hormone (LH), follicle stimulating hormone (FSH) and testosterone.

### Spermatozoa density and motility

Immediately after sacrifice, right and left cauda epididymides of each animal were separately minced and thoroughly mixed in 10 ml of warm (36 °C) 0.9% NaCl. 20 μl of the mixture were transferred to a Malassez haemocytometer and observed under a light microscope (OLYMPUS, X40). Motile and non-motile spermatozoa were counted in 10 fields and the percentage of motile spermatozoa was determined [29, 37].

For spermatozoa density, a twenty-fold dilution was made by mixing the spermatozoa suspension with 0.9% NaCl solution. After shaking, 20 μl of the mixture was transferred to a Malassez haemocytometer, observed under a light microscope (OLYMPUS, X40) and, spermatozoa were counted in 10 fields [37].

### Spermatozoa viability and morphology

To determine spermatozoa viability, spermatozoa suspension (10 μl) was mixed with eosin (10 μl at 1%) and nigrosin (30 μl at 5%) on a slide. The mixture was smeared on the slide and examined under a light microscope (OLYMPUS, 40X). Ten (10) fields on the slide were selected in order to appreciate spermatozoa that were stained pink or red (considered dead), and the unstained spermatozoa (considered viable). The percentage of spermatozoa viability was expressed [37].

The spermatozoa morphology was determined using eosin/nigrosin staining. Eosin (10 μl at 1%) and nigrosin (30 μl at 5%) were added to 10 μl of spermatozoa suspension. The prepared smear was used after incubation for 5 min in an oven (45 °C). Ten (10) fields on the slide were selected in order to appreciate various abnormalities of spermatozoa (head and tail abnormalities, cytoplasmic droplets, tailless spermatozoa) as described previously [37].

### Oxidative stress markers and sex hormones

Testis was crushed in a mortar containing Tris buffer so as to obtain a 10% homogenate. Supernatant was collected from homogenate and centrifuged (3000 x g for 10 min). The supernatant was used for protein, MDA, SOD and catalase analysis. Proteins were measured using a commercial kit (Roche diagnostics cobas c-1111) and protocols were performed according to the manufacturer’s instructions. The MDA content was measured using thiobarbituric acid reaction [38]. The tissue SOD and catalase activities were evaluated as described by Dimo et al. [39]. Plasmatic LH, FSH and testosterone levels were quantified using ELISA methods in conformity with commercial kit instructions (Accubind, Monobind. Lake Forest, USA) [40, 41].

### Statistical analysis

Data were expressed as mean ± S.E.M. One-way analysis of variance (ANOVA) followed by Tukey-HSD post-hoc test was used to determine statistical differences among groups. All analyses were performed using Statistica software (version 8.0, StatSoft, Inc., Tulsa, USA). Significance level was set at *p* < 0.05.

## Results

### Effect of different treatments on the relative weight of testes and epididymides

**Fig. 2:**
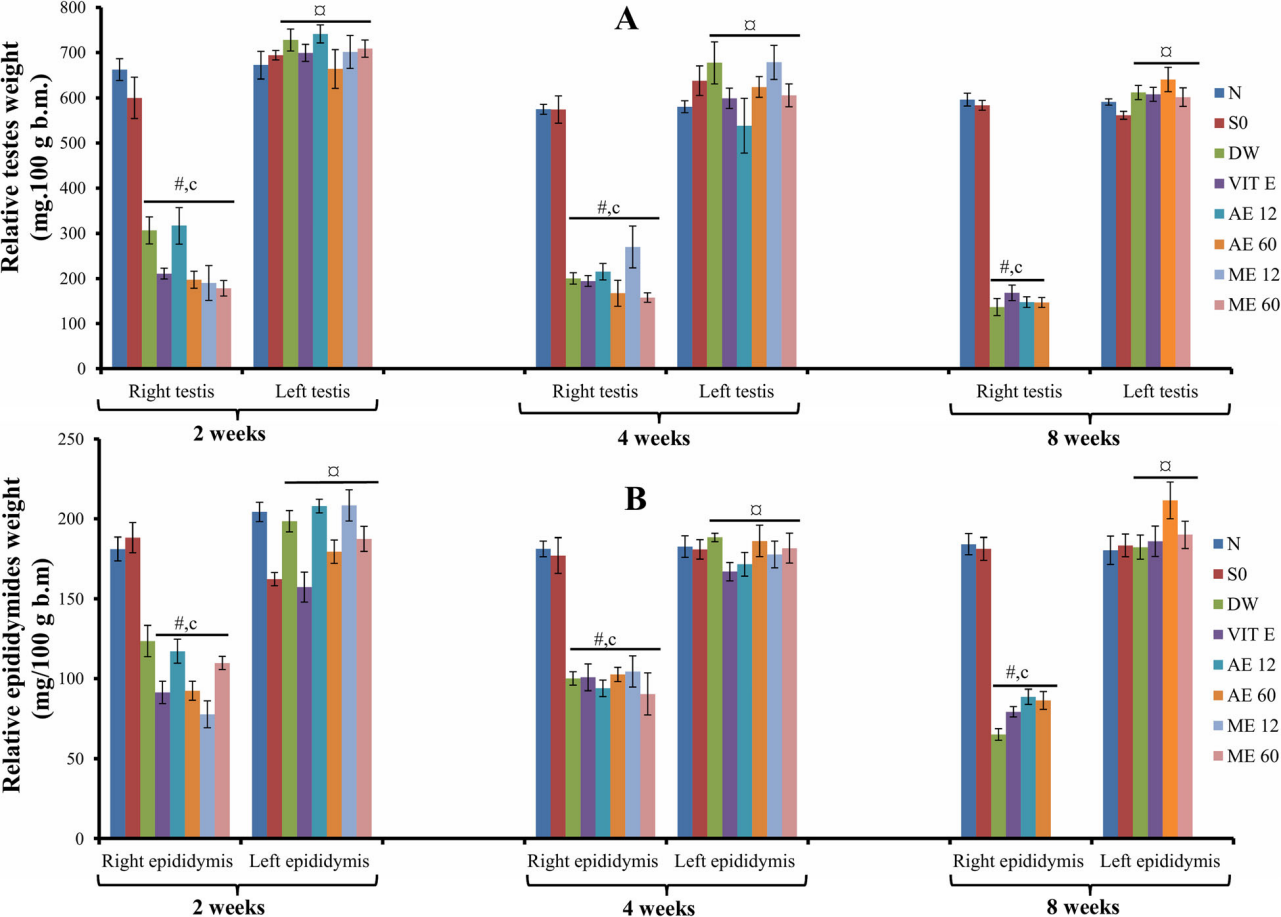
Effects of different treatments on the relative weights of testes (**a**) and epididymides (**b**)*.* Values are mean ± SEM. *: *p* < 0.05; Number of rats per group = 5.c: *p* < 0.001 compared to sham operated; #: *p* < 0.001 compared to normal; ¤: *p* < 0.001 compared to right testis (same group). Abbreviations: N: normal; SO: sham operated; DW: distilled water; Vit E: vitamin E; AE12 / AE60: aqueous extract administered at 12 mg/kg or 60 mg/kg; ME12 / ME60: methanolic extract administered at 12 mg/kg or 60 mg/kg

The right testis and epididymis weights were significantly (*p* < 0.001) lowered after induction of cryptorchidism, compared to sham operated and normal groups. In each cryptorchid animal administered with vitamin E or plant extracts, the testis and epididymal weights were significantly increased compared to the right cryptorchid side (Fig. 2a, b).

### Effect of different treatments on spermatozoa parameters

In the right testis, cryptorchidism induced azoospermia while no significant change was observed in the contralateral testis. Therefore, spermatozoa motility, viability and normality assessment in the right epididymis of cryptorchid animals were impossible.

Azoospermia persisted in the right epididymis of cryptorchid rats after 2, 4 and 8 weeks of treatment with the vehicle (distilled water) or dugs (vitamin E and plant extracts). In the left contralateral epididymis, *R. Apetalus* significantly (*p* < 0.05–0.001) increased the spermatozoa motility (except aqueous extract at 12 mg/kg) and spermatozoa with normal morphology (both extracts) after 2, 4 and 8 weeks of treatment. *R. Apetalus* also increased (*p* < 0.05–0.001) the spermatozoa viability (4 weeks: methanolic extract at 60 mg/kg; 8 weeks: aqueous and methanolic extracts at 60 mg/kg) and spermatozoa count (4 weeks: methanolic extract at 12 and 60 mg/kg; 8 weeks: aqueous and methanolic extracts at 60 mg/kg) compared to untreated cryptorchid rats (DW group). However, at all treatment periods, the methanolic extract (60 mg/kg) was more effective than the aqueous extract (Table 1).

**Table 1:** Effects of different treatments on sperm parameters

Sperm parameters								
Groups	Viability (%)		Sperm count (10^**6**^/mL)		Motility (%)		Normality (%)	
	Right testis	Left testis	Right testis	Left testis	Right testis	Left testis	Right testis	Left testis
**2 weeks**								
**N**	92.03 ± 0.5	91.03 ± 0.6	178.62 ± 7.00	179.62 ± 8.00	60.9 ± 4.05	61.06 ± 4.65	70.50 ± 0.87	68.07 ± 0.94
**SO**	89.26 ± 0.57	88.16 ± 0.68	113.30 ± 11.75	114.50 ± 12.45	66.05 ± 2.59	67.55 ± 3.49	65.56 ± 0.96	64.16 ± 1.64
**DW**	0#, c,¤^,¤^	93.50 ± 1.42	0#, c,¤^,¤^	92.12 ± 27.29	0#, c,¤	44.37 ± 0.83	0#, c,¤^,¤^	50.85 ± 2.28
**VIT E**	0#, c,¤^,¤^	87.36 ± 2.47	0#, c,¤^,¤^	133.75 ± 15.45	0#, c,¤^,¤^	58.02 ± 7.49	0#, c,¤^,¤^	59.7 ± 2.66^*^
**AE 12**	0#, c,¤^¤^	91.81 ± 1.60	0#, c,¤^,¤^	111.75 ± 17.44	0#, c,¤^,¤^	65.47 ± 5.85	0#, c,¤^,¤^	70.95 ± 1.74^***^
**AE 60**	0#, c,¤^,¤^	91.90 ± 3.53	0#, c,¤^,¤^	107.75 ± 13.58	0#, c,¤^,¤^	72.08 ± 4.17^*^	0#, c,¤^,¤^	71.11 ± 1.61^***^
**ME 12**	0#, c,¤^,¤^	90.10 ± 1.50	0#, c,¤^,¤^	61.12 ± 5.92	0#, c,¤^,¤^	75.34 ± 5.75^*^	0#, c,¤^,¤^	62.69 ± 1.08^***^
**ME 60**	0#, c,¤^,¤^	98.90 ± 1.86	0#, c,¤^,¤^	63.50 ± 4.93	0#, c,¤^,¤^	84.01 ± 2.08^***^	0#, c,¤^,¤^	75.04 ± 1.33^***^
**4 weeks**								
**N**	93.14 ± 0.67	91.03 ± 0.67	156.62 ± 7.50	157.87 ± 7.41	70.9 ± 3.85	71.84 ± 1.42	{66.13 ± 0.87)	67.35 ± 0.72
**SO**	87.56 ± 0.46	88.16 ± 0.68	151.20 ± 9.75	153.12 ± 3.47	75.05 ± 2.35	77.43 ± 2.08	65.06 ± 1.22	65.46 ± 1.24
**DW**	0#, c,¤	81.34 ± 1.85	0#, c,¤	88.50 ± 10.87	0#, c,¤	65.49 ± 1.68	0#, c,¤	53.45 ± 1.29
**VIT E**	0#, c,¤	87.17 ± 0.70	0#, c,¤	108.38 ± 12.88	0#, c,¤	74.41 ± 1.38	0#, c,¤	64.8 ± 2.78^*^
**AE 12**	0#, c,¤	84.95 ± 0.83	0#, c,¤	74.88 ± 9.92	0#, c,¤	72.91 ± 2.61]	0#, c,¤	72.54 ± 1.21^***^
**AE 60**	0#, c,¤	86.02 ± 1.58	0#, c,¤	102.50 ± 7.23	0#, c,¤	82.93 ± 3.06^**^	0#, c,¤	78.54 ± 1.82^***^
**ME 12**	0#, c,¤	84.01 ± 0.91	0#, c,¤	135.13 ± 24.95^*,Δ^	0#, c,¤	80.39 ± 2.90^*^	0#, c,¤^, ¤^	51.71 ± 1.91^***^
**ME 60**	0#, c,¤	91.34 ± 4.16^**^	0#, c,¤	144.00 ± 28.80^*,Δ^	0#, c,¤	85.67 ± 1.76^***^	0#, c,¤	79.84 ± 1.41^***^
**8 weeks**								
**N**	92.12 ± 0.65	84.47 ± 2.94	184.65 ± 7.45	183 ± 7.29	84.47 ± 2.94	84.47 ± 2.94	67.94 ± 0.94	66.32 ± 0.81
**SO**	89.25 ± 0.75	89.83 ± 1.76	86.92 ± 3.86	185 ± 3.96	89.83 ± 1.76	89.83 ± 1.76	63.81 ± 0.87	64.16 ± 1.64
**DW**	0#, c,¤	72.92 ± 2.62	0#, c,¤	106.66 ± 6.08	0#, c,¤	72.92 ± 2.61	0#, c,¤	50.85 ± 2.28
**VIT E**	0#, c,¤	81.97 ± 3.05	0#, c,¤	150.62 ± 7.67^*^	0#, c,¤	81.97 ± 3.05	0#, c,¤	59.7 ± 2.66^*^
**AE 60**	0#, c,¤	85.59 ± 3.96^*^	0#, c,¤	176.35 ± 4.03^**, Δ,^•	0#, c,¤	85.59 ± 3.96^*^	0#, c,¤	77.20 ± 2.62^***^
**ME 60**	0#, c,¤	86.54 ± 2.37^*^	0#, c,¤	147.50 ± 5.90^**,Δ,^•	0#, c,¤	86.54 ± 2.37^*^	0#, c,¤	79.75 ± 1.33^***^

All values are expressed as mean ± SEM. Number of rat per group = 5

*N* normal, *SO* sham operated, *DW* distilled water, *VIT E* vitamin E, *AE* aqueous extract, *ME* methanolic extract

#: *p* < 0.001 compared to normal; c: *p* < 0.001 compared to sham operated; *p<0.05;**p<0.01;***p<0.001 compared to DW; ¤: *p* < 0.001 compared to left testis (same group); Δ*p* < 0.001 compared to week 2 (same group); •*p* < 0.001 compared to week 4 (same group)

### Effect of different treatments on spermatozoa morphological abnormalities

No spermatozoa was observed from the cryptorchid testis, whereas from the contralateral testis, an increase in the percentage of sperm with abnormal head, abnormal tail, tailless head and cytoplasmic droplet was recorded when compared to normal and sham operated.

When compared with the untreated cryptorchid rats (DW group), administration of vitamin E or *R. Apetalus* was followed by a significantly (*p* < 0.001) decrease in the percentage of spermatozoa with abnormal head and abnormal tail after 2, 4 and 8 weeks of treatment. Moreover, *R. apetalus* significantly decreased the percentage of cytoplasmic droplet after 2 weeks (methanolic extract at 60 mg/kg), 4 weeks (methanolic extract at 60 mg/kg) and 8 weeks (aqueous and methanolic extracts at 60 mg/kg) of treatment. The effect of *R. Apetalus* was dose-dependent (Table 2).

**Table 2:** Effects of different treatments on sperm abnormalities

Sperm morphological abnormalities								
Groups	Abnormal head (%)		Abnormal tail (%)		Tailless head (%)		Cytoplasmic droplet (%)	
	Right testis	Left testis	Right testis	Left testis	Right testis	Left testis	Right testis	Left testis
**2 weeks**								
**N**	4.11 ± 0.59	3.86 ± 0.40	3.73 ± 0.36	3.52 ± 0.26	3.88 ± 0.58	3.81 ± 0.86	4.3 ± 0.90	4.39 ± 1.19
**SO**	3.84 ± 0.46	3.48 ± 0.36	4.07 ± 0.50	4.87 ± 0.40]	3.28 ± 0.69	3.30 ± 1.62	2.72 ± 0.91	2.43 ± 1.08
**DW**	0^#, c, ¤^	21.41 ± 1.79^#, c^	0^#, c, ¤^	21.17 ± 3.30^#, c^	0^#, c, ¤^	7.56 ± 1.43^#, c^	0^#, c, ¤^	9.44 ± 0.61^#, c^
**VIT E**	0^#, c,¤^	4.71 ± 0.62^***^	0^#, c, ¤^	5.90 ± 0.62^***^	0^#, c, ¤^	5.29 ± 1.27	0^#, c, ¤^	5.29 ± 1.27
**AE 12**	0^#, c, ¤^	10.01 ± 2.59^***^	0^#, c, ¤^	12.32 ± 1.77^*^	0^#, c, ¤^	7.30 ± 1.58	0^#, c, ¤^	6.33 ± 1.33
**AE 60**	0^#, c, ¤^	7.34 ± 0.36^***^	0^#, c, ¤^	8.49 ± 1.89^***^	0^#, c, ¤^	5.98 ± 1.63	0^#, c, ¤^	6.31 ± 0.89
**ME 12**	0^#, c, ¤^	11.12 ± 0.59^***^	0^#, c, ¤^	15.59 ± 1.54^*^	0^#, c, ¤^	6.41 ± 1.73	0^#, c, ¤^	6.19 ± 0.5
**ME 60**	0^#, c, ¤^	6.93 ± 1.13^***^	0^#, c, ¤^	11.91 ± 0.57^**^	0^#, c, ¤^	4.17 ± 0.40	0^#, c, ¤^	3.74 ± 0.75^**^
**4 weeks**								
**N**	3.22 ± 0.42	3.96 ± 0.45	3.97 ± 0.46	3.42 ± 0.47	3.25 ± 0.94	3.70 ± 0.61	4.18 ± 1.04	4.74 ± 0.99
**SO**	3.28 ± 0.27	3.98 ± 0.51	4.28 ± 0.31	5.02 ± 0.53	4.91 ± 0.92	3.58 ± 0.83	3.56 ± 0.77	3.13 ± 0.76
**DW**	0^#, c, ¤^	23.51 ± 1.29^#, c^	0^#, c, ¤^	22.15 ± 2.30^#, c^	0^#, c, ¤^	8.34 ± 0.94^#, c^	0^#, c, ¤^	10.14 ± 1.50^#, c^
**VIT E**	0^#, c, ¤^	6.80 ± 0.52^***^	0^#, c, ¤^	4.30 ± 0.42^***^	0^#, c, ¤^	6.19 ± 1.40	0^#, c, ¤^	6.22 ± 1.37
**AE 12**	0^#, c, ¤^	12.04 ± 1.34^***^	0^#, c, ¤^	11.52 ± 1.86^*^	0^#, c, ¤^	8.91 ± 1.24	0^#, c, ¤^	7.66 ± 1.91
**AE 60**	0^#, c, ¤^	5.25 ± 0.47^***^	0^#, c, ¤^	7.57 ± 1.32^***^	0^#, c, ¤^	4.80 ± 1.52	0^#, c, ¤^	5.10 ± 0.54
**ME 12**	0^#, c, ¤^	12.42 ± 0.67^***^	0^#, c, ¤^	14.09 ± 1.64^*^	0^#, c, ¤^	7.48 ± 0.97	0^#, c, ¤^	8.21 ± 0.75
**ME 60**	0^#, c, ¤^	5.99 ± 0.97^***^	0^#, c, ¤^	10.82 ± 0.68^**^	0^#, c, ¤^	4.65 ± 0.49	0^#, c, ¤^	3.14 ± 0.86^**^
**8 weeks**								
**N**	4.25 ± 0.45	4.26 ± 0.56	3.92 ± 0.32	3.17 ± 0.16	3.70 ± 0.46	3.95 ± 0.26	5.09 ± 0.75	5.49 ± 1.09
**SO**	3.52 ± 0.41	3.47 ± 0.30	3.96 ± 0.39	3.87 ± 0.50	6.41 ± 0.88	5.07 ± 0.94	4.73 ± 0.39	3.85 ± 0.45
**DW**	0^#, c, ¤^	22.47 ± 1.39^#, c^	0^#, c, ¤^	21.17 ± 3.35^#, c^	0^#, c, ¤^	8.56 ± 1.43^#, c^	0^#, c, ¤^	9.56 ± 0.66^#, c^
**VIT E**	0^#, c, ¤^	4.70 ± 0.61^***^	0^#, c, ¤^	5.90 ± 0.98^***^	0^#, c, ¤^	5.29 ± 1.27	0^#, c, ¤^	5.29 ± 1.27
**AE 60**	0^#, c, ¤^	7.6 ± 2.15^***^	0^#, c, ¤^	9.40 ± 2.20^**^	0^#, c, ¤^	7.60 ± 1.17	0^#, c, ¤^	4.40 ± 0.75^**^
**ME 60**	0^#, c, ¤^	5.55 ± 1.44^***^	0^#, c, ¤^	9.63 ± 1.55^**^	0^#, c, ¤^	7.18 ± 0.55	0^#, c, ¤^	1.18 ± 0.59^***^

All values are expressed as mean ± SEM. Number of rats per group = 5

*N* normal, *SO* sham operated, *DW* distilled water, *VIT E* vitamin E, *AE* aqueous extract, *ME* methanolic extract

#: *p* < 0.001 compared to normal; c: *p* < 0.001compared to sham operated; *p<0.05; **p<0.01; ***p<0.001 compared to DW; ¤: *p* < 0.001 compared to left testis (same group)

### Effect of different treatments on testicular proteins

**Fig. 3:**
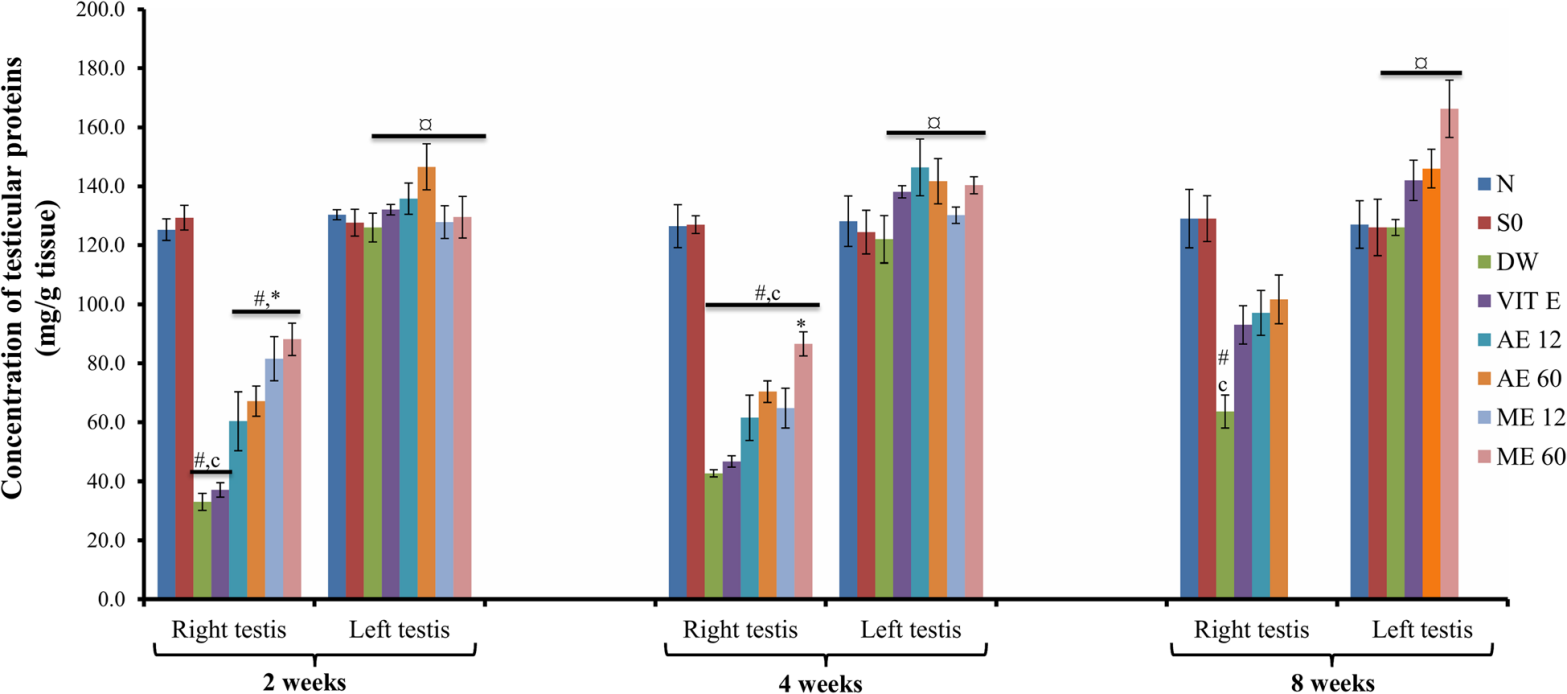
Effects of different treatments on testicular protein levels*.* Values are mean ± SEM. Number of rats per group = 5. c: *p* < 0.001 compared to sham operated;#: *p* < 0.001 compared to normal;*p<0.001 compared to DW;¤: *p* < 0.001 compared to right testis (same group);Abbreviations: N: normal; SO: sham operated; DW: distilled water; Vit E: vitamin E; AE12 / AE60: aqueous extract administered at 12 mg/kg or 60 mg/kg; ME12 / ME60: methanolic extract administered at 12 mg/kg or 60 mg/kg

In all untreated cryptorchid rats, the protein concentrations were lowered in the right testis compared to the left testis. Vitamin E and *R. apetalus* extracts significantly (*p*<0.05) increased the testicular protein contents (in the right testis) after 2 weeks of treatment. At all treatment periods, the highest dose of plant extracts (60 mg/kg) was the most effective in the cryptorchid testis (Fig. 3).

### Effect of different treatments on oxidative stress markers

The occurrence of cryptorchism was associated with oxidative stress characterized in the right testis by a significant decrease (*p* < 0.05) in catalase and SOD activities and a high lipid peroxidation (increase in MDA).

**Fig. 4:**
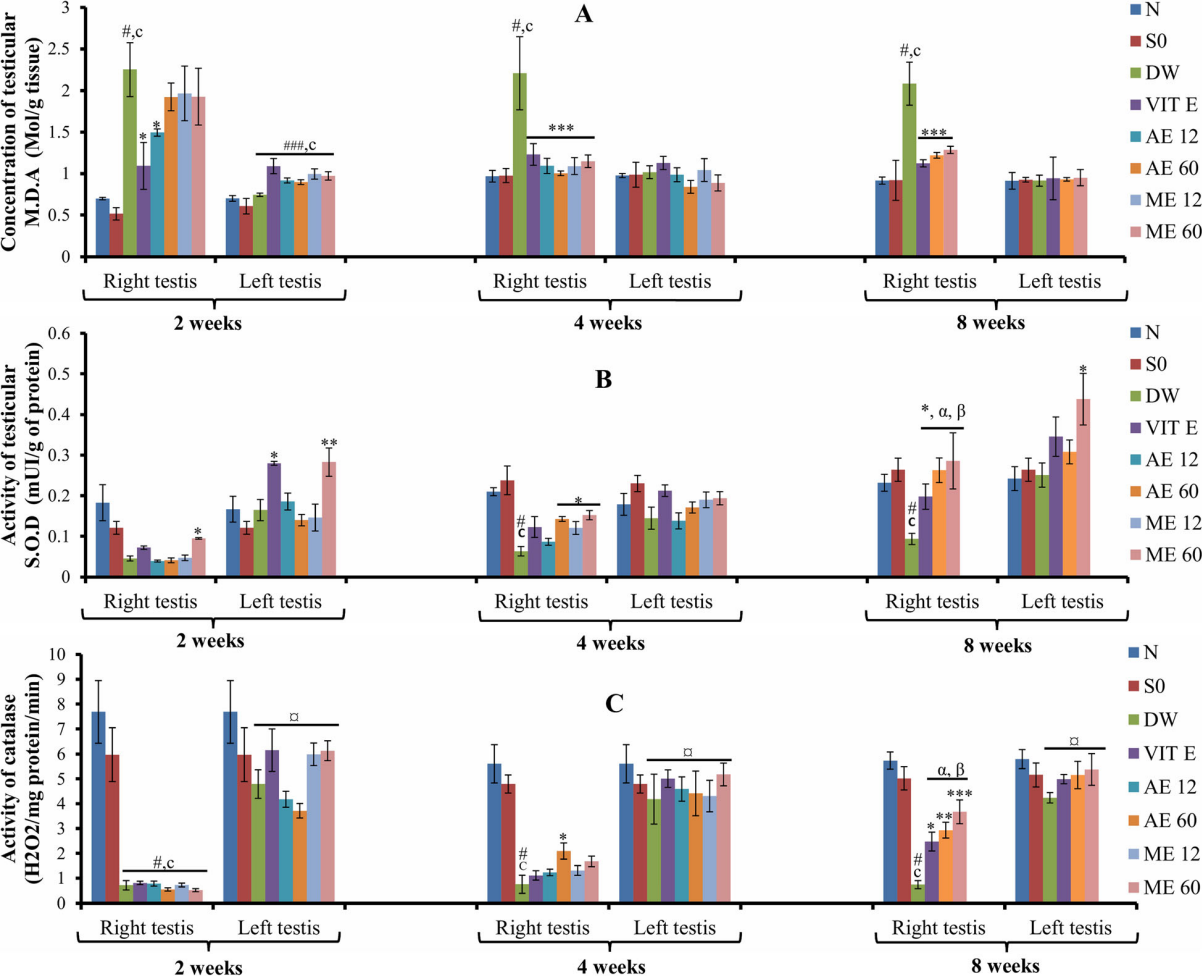
Effects of different treatments on testicular MDA (**a**), SOD (**b**) and catalase (**c**) activities. Values are mean ± SEM. Number of rats per group = 5.c: *p* < 0.001 compared to sham operated;#: *p* < 0.001 compared to normal;*: *p*<0.05;**: *p*<0.01***: *p*<0.001 compared to DW;¤: *p* < 0.001 compared to right testis (same group); α: *p* < 0.001 compared to 2 weeks (same group); β: *p* < 0.001 compared to 4 weeks (same group). Abbreviations: MDA: malondialdehyde; SOD: superoxide dismutase; N: normal; SO: sham operated; DW: distilled water; Vit E: vitamin E; AE12 / AE60: aqueous extract administered at 12 mg/kg or 60 mg/kg; ME12 / ME60: methanolic extract administered at 12 mg/kg or 60 mg/kg

Vitamin E and *R. apetalus* significantly (*p* < 0.05) decreased MDA level in the right testis after 2 weeks (aqueous extract at 12 mg/kg), 4 weeks (aqueous and methanolic extracts at 12 mg/kg and 60 mg/kg) and 8 weeks (aqueous and methanolic extracts at 60 mg/kg) of treatment, compared to the untreated cryptorchid rats (Fig. 4a).

SOD activity in the right testis was significantly (*p* < 0.05) elevated in rats given *R. apetalus* for 2 weeks (aqueous extract at 60 mg/kg), 4 weeks (aqueous extract at 60 mg/kg and methanolic extract at 12 mg/kg and 60 mg/kg) and 8 weeks (aqueous and methanolic extracts at 60 mg/kg), compared to the untreated cryptorchid group. In the left testis, SOD activity was also increased (*p* < 0.05–0.01) in rats treated with vitamin E (2 weeks) and methanolic extract of *R. apetalus* (60 mg/kg; 2 and 8 weeks) compared to DW group. In both testes, the effects of vitamin E and *R. Apetalus* were more pronounced after 8 weeks of treatment (Fig. 4b).

The decrease observed in catalase activity in the cryptorchid testis was reversed in a dose-dependent manner after vitamin E and *R. apetalus* treatments (Fig. 4c).

### Effect of different treatments on sex hormones

**Fig. 5:**
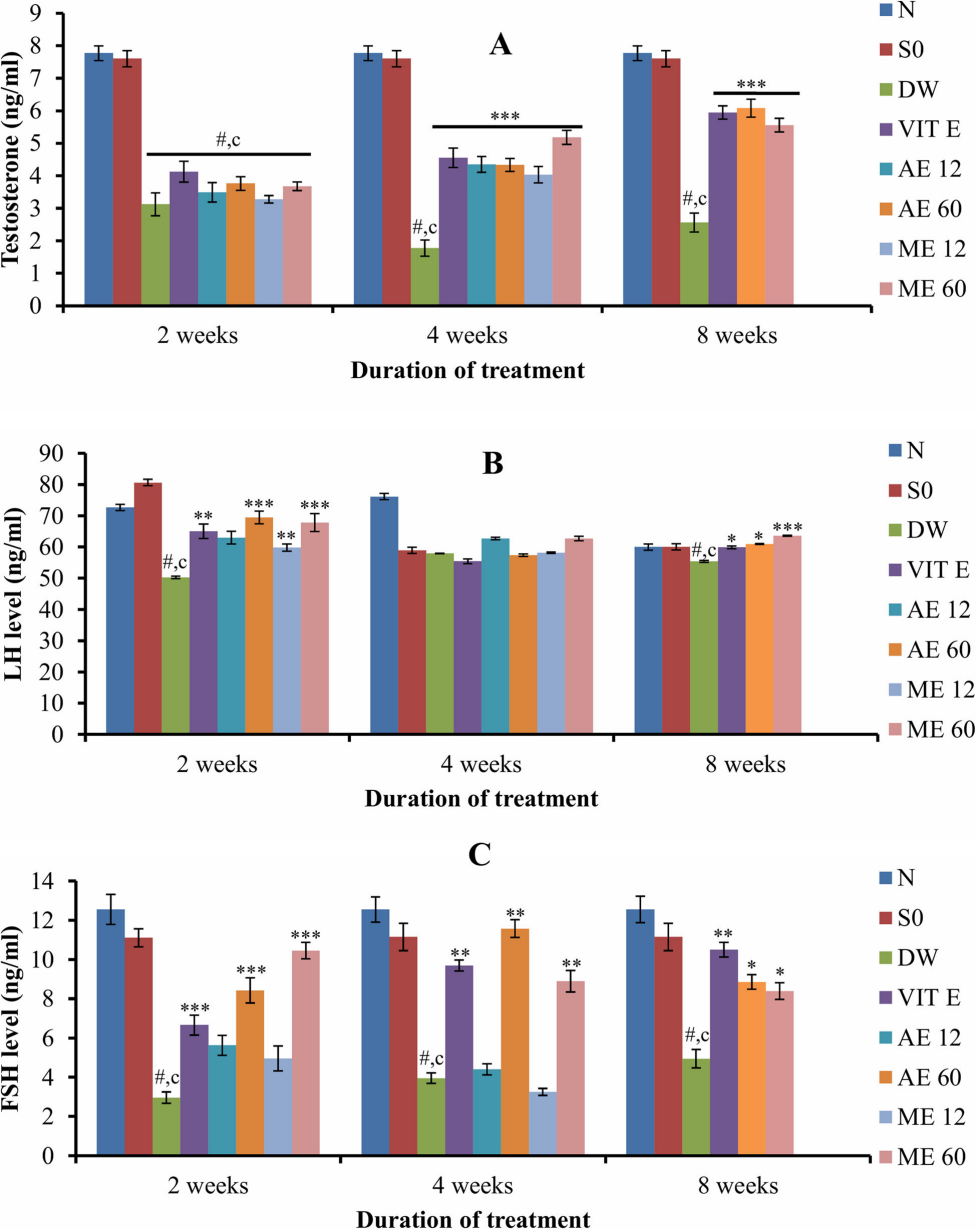
Effects of different treatments on plasmatic testosterone (**a**), LH (**b**) and FSH (**c**). Values are mean ± SEM. Number of rats per group = 5. c: *p* < 0.001 compared to sham operated; #: *p* < 0.001 compared to normal;*: *p*<0.05;**: *p*<0.01***: *p*<0.001 compared to DW. Abbreviations: N: normal; SO: sham operated; DW: distilled water; Vit E: vitamin E; AE12 / AE60: aqueous extract administered at 12 mg/kg or 60 mg/kg; ME12 / ME60: methanolic extract administered at 12 mg/kg or 60 mg/kg

Plasmatic testosterone, LH and FSH levels were significantly (*p* < 0.001) decreased after cryptorchidism set up. Vitamin E and *R. apetalus* extracts elevated the plasmatic testosterone level in a time-dependent manner. In the cryptorchid rats, the highest value of plasmatic testosterone was observed in animals administered for 8 weeks with the aqueous extract of *R. apetalus* (60 mg/kg) (Fig. 5a). Compared to the DW group, we noticed that the plasmatic LH level was significantly (*p* < 0.01–0.001) increased in rats treated for 2 and 8 weeks with vitamin E and *R. apetalus* (both extracts at all doses) (Fig. 5b). *R. apetalus* also increased (*p* < 0.05–0.001) the plasmatic FSH level (in a dose-dependent manner) after 2, 4 and 8 weeks of treatment. In rats given vitamin E, the plasmatic FSH level increased gradually (*p* < 0.01–0.001) from week 2 to week 8 (Fig. 5c).

### Effect of different treatments on morphometric characteristics of testis

Cryptorchidism significantly (*p* < 0.001) decreased the diameter of the seminiferous tubes and seminiferous epithelium thickness, and increased peri-vascular fibrosis in the right testis, but did not affect the left testis. Vitamin E significantly (*p* < 0.05–0.001) increased the diameter of the seminiferous tubes (both testis: weeks 4 and 8) and seminiferous epithelium thickness (left testis: weeks 2 and 8), and decreased the peri-vascular fibrosis (right testis: weeks 2, 4 and 8) compared to DW group. Similarly, *R. apetalus* (all doses) increased (*p* < 0.05–0.001) the diameter of the seminiferous tubes and seminiferous epithelium thickness, but decreased (*p* < 0.05–0.001) the peri-vascular fibrosis in the right testis after 2, 4 and 8 weeks of treatment (Table 3).

**Table 3:** Effect of different treatments on the diameter of the seminiferous tubes, seminiferous epithelium thickness and peri-vascular fibrosis

Groups	Diameter of the seminiferous tubes (μm)		Germ cell thickness (μm)	Peri-vascular fibrosis (μm)		
	Right testis	Left testis	Right testis	Left testis	Right testis	Left testis
**2 weeks**						
**N**	653.63 ± 12.30	656.73 ± 12.73	229.87 ± 7.07	234.35 ± 5.24	16.63 ± 1.82	16.12 ± 1.81
**SO**	652.21 ± 12.30	662.39 ± 10.23	230.86 ± 8.72	238.20 ± 8.78	16.41 ± 1.87	16.52 ± 1.72
**DW**	436.64 ± 12.89^#,c**,¤**^	663.31 ± 10.80	0 ^#, c**, ¤**^	234.26 ± 6.46	48.30 ± 2.76 ^#, c, ¤^	16.87 ± 2.31
**VIT E**	461.91 ± 12.30	683.26 ± 11.63	0 ^#, c**, ¤**^	277.43 ± 10.08^**^	33.91 ± 1.41^***^	14.52 ± 1.03
**AE 12**	441.76 ± 9.76	681.67 ± 8.80	0 ^#, c**, ¤**^	331.69 ± 8.88^***^	28.06 ± 2.87^***^	13.33 ± 1.00
**AE 60**	481.91 ± 8.05^**^	696.91 ± 8.85	0 ^#, c**, ¤**^	331.21 ± 11.59^***^	27.76 ± 2.03^***^	12.46 ± 0.91
**ME 12**	480.46 ± 9.18^***^	667.91 ± 10.37	0 ^#, c**, ¤**^	358.79 ± 12.71^***^	25.01 ± 0.95^***^	14.60 ± 1.40
**ME 60**	498.59 ± 9.49^***^	669.76 ± 12.89	0 ^#, c**, ¤**^	373.02 ± 12.91^***^	24.94 ± 1.17^***^	11.46 ± 1.19
**4 weeks**						
**N**	659.93 ± 9.32	658.1 ± 7.52	228.83 ± 8.50	236.49 ± 9.39	15.65 ± 2.650	16.58 ± 2.68
**SO**	655.41 ± 12.09	661.36 ± 11.68	229.41 ± 9.13	240.87 ± 10.48	16.64 ± 1.31	16.30 ± 1.01
**DW**	387.51 ± 7.37^#, c**, ¤**^	658.29 ± 9.76	0^#, c**, ¤**^	227.03 ± 7.29	40.10 ± 3.26^#, c**, ¤**^	16.02 ± 1.02
**VIT E**	476.63 ± 11.65^***^	674.2 ± 12.97^*^	0^#, c**, ¤**^	273.39 ± 6.41	34.98 ± 1.09^***^	16.40 ± 0.63
**AE 12**	444.09 ± 9.03^***^	690.06 ± 6.86	0^#, c**, ¤**^	335.89 ± 11.20^*^	27.58 ± 0.95^***^	12.03 ± 2.23
**AE 60**	464.37 ± 12.63^***^	721.57 ± 11.76	0^#, c**, ¤**^	382.97 ± 12.14^*^	26.74 ± 1.06^***^	12.01 ± 0.95
**ME 12**	446.34 ± 12.46^***^	668.67 ± 12.637	0^#, c,**, ¤**^	380.61 ± 9.22	{24.27 ± 2.16^***^	16.61 ± 0.93
**ME 60**	481.49 ± 10.19^***^	707.69 ± 11.54	0^#, c**, ¤**^	383.66 ± 10.85^*^	20.16 ± 1.68^***^	13.34 ± 1.15
**8 weeks**						
**N**	662.31 ± 9.46	659.86 ± 7.65	241.26 ± 6.65	244.38 ± 7.87	17.20 ± 1.73	17.17 ± 1.59
**SO**	630.13 ± 4.30	661.63 ± 7.31	236.36 ± 4.50	237.20 ± 4.66	16.27 ± 1.23	16.21 ± 1.35
**DW**	363.46 ± 9.73^#, c, ¤^	663.89 ± 10.90	0^#, c, ¤^	234.83 ± 7.48	45.29 ± 3.84^#, c, ¤^	16.33 ± 1.63
**VIT E**	470.23 ± 10.45^***^	667.16 ± 8.36	0^#, c, ¤^	279.73 ± 7.37^***^	34.53 ± 1.54^***^	16.82 ± 1.38
**AE 60**	491.01 ± 5.56^***^	683.04 ± 6.33	0^#, c, ¤^	411.59 ± 3.81^***^	23.90 ± 1.76^***^	12.89 ± 0.97
**ME 60**	495.87 ± 8.04^***^	740.66 ± 9.71	0^#, c, ¤^	428.69 ± 7.48^***^	29.60 ± 1.30^***^	13.94 ± 1.16

All values are expressed as mean ± SEM. Number of rats per group = 5

*Abbreviations*: *N* normal, *SO* sham operated, *DW* distilled water, *VIT E* vitamin E, *AE* aqueous extract, *ME* methanolic extract

#: *p* < 0.001 compared to normal; c: *p* < 0.001 compared to sham operated; *p<0.05; **p<0.01; ***p<0.001 compared to DW; ¤: *p* < 0.001 compared to left testis (same group); ¤: *p* < 0.001 compared to left testis (same group)

### Effect of different treatments on fertility potential

The fertility potential of cryptorchid rats was evaluated using females with proven fertility. A decrease in gestation index (62.5%) and fertility index (50.00%) was observed when compared to normal and sham operated animals. After vitamin E and *R. apetalus* treatments, an improvement of gestation index and fertility index was recorded. The methanolic extract of *R. apetalus* was the most active extract (Table 4).

**Table 4:** Effects of different treatments on fertilization potential

Groups	Number of females mated	Number of females with a positive vaginal smear	Number of pregnant females	Gestation index (%)	Fertility index (%)	Average litter size
**N**	10	9	9	90.00	100.00	6.67 ± 0.34
**SO**	10	9	8	88.00	80.00	6.44 ± 0.37
**DW**	10	8	5	62.5	50.00	2.90 ± 0.35
**VIT E**	10	9	8	88.00	80.00	4.9 ± 0.043
**AE 60**	10	10	9	90.00	90.00	6.50 ± 0.5
**ME 60**	10	9	10	100.00	100.00	6.80 ± 0.66

*Abbreviations*: *N* normal, *SO* sham operated, *DW* distilled water, *Vit E* vitamin E, *AE* aqueous extract, *ME* methanolic extract

## Discussion

Cryptorchidism is an endocrinopathy considered as an important cause of male infertility [42]. The current study evaluated the effects of aqueous and methanolic extracts of *R. apetalus* on various reproductive (organ weights, spermatozoa parameters, testicular proteins, sex hormones, fertility potential and morphometric characteristics of testis) and metabolic (oxidative stress markers) parameters in unilateral induced cryptorchid rats. Results from this study showed that *R. apetalus* improved the fertility potential of cryptorchid rats by increasing the relative weight of testes and epididymis and, alleviating spermatozoa characteristics, testicular proteins and sex hormones in one hand, and by reducing the oxidative stress in the testis through a significant decrease in MDA and a high SOD and catalase activities in the other hand.

CPT can experimentally be induced by several techniques including surgery, hormone application or transgenic methods [32, 33]. After induction, the testicular weight decreases gradually due to hormonal depletion, with atrophy and few germinal cells, which negatively affect steroidogenesis and spermatogenesis [33, 43]. In this study, the surgical CPT model was chosen. In rats; this model induces a CPT similar to human CPT. We noticed that all rats developed unilateral cryptorchidism after induction, as also reported by Muhammetnur et al. [18].

Testicular and epididymal weights are good indicators of cryptorchidism. As reported by Muhammetnur et al. [18], the relative weights of testes and epididymis were significantly lowered in cryptorchid animals, compared with control. Indeed, cryptorchidism is associated with lipid peroxidation and oxidative stress, characterized by an excessive production of reactive oxygen species (ROS), which induce apoptosis in the testes and cause degeneration of the germinal epithelium of seminiferous tubules, leading to low testicular weight [44]. The decrease in epididymal weight may result from a quantitatively reduced spermatogenesis (as indicated by azoospermia). In each cryptorchid rat given vitamin E or plant extracts, the left testicular and epididymal weights were significantly increased compared to the right side. This result was expected because of the cryptorchidism condition of the right epididymis. Similar findings were reported by Muhammetnur et al. [18].

Cryptorchidism seriously damaged steroidogenesis and spermatogenesis. In the right epididymis, no spermatozoa was found whereas spermatozoa morphology abnormalities (abnormal head, abnormal tail, tailless head and cytoplasmic droplet) were elevated in the contralateral epididymis, compared with normal and sham operated [17, 44]. These abnormal spermatozoa characteristics could be due to the adverse effects of the thermal stress and oxidative stress, which negatively affect spermatogenesis and steroidogenesis. Indeed, under cryptorchidism condition, spermatozoa which are released in the germinal epithelium carry more residual cytoplasm and are then considered as abnormal (defective) [45]. However, *R. apetalus* improved spermatozoa characteristics with a remarkable effect (at all treatment periods) observed in rats administered with the methanolic extract (60 mg/kg). The effectiveness of methanolic extract in improving the spermatozoa characteristics could be correlated with its ability to easily extract antioxidant compounds such as saponins, alkaloids, flavonoids, tannins, triterpenoids and vitamins E and C [23] and, improve sex hormone levels. Similar results were reported in cryptorchid rats administered with *Moringa oleifera* [17].

Oxidative stress is considered as an important cause of spermatozoa abnormalities in cryptorchid individuals [46]. It has been shown that high scrotal temperature increases the production of heat shock proteins and free radicals, which negatively affect spermatogenesis, by damaging the purine and pyrimidine bases, leading to abnormal spermatozoa production [18]. Low testicular protein activities as well as an increase in malondialdehyde (MDA) clearly indicate lipid peroxidation. In the present work, cryptorchidism was characterized by a high MDA concentration, low antioxidant enzyme (SOD and catalase) activities and a decrease in testicular protein levels. High testicular temperature promotes ROS overproduction and peroxidation of the spermatozoa membrane, leading to the death of germ cells [47, 48]. Wdowiak et al. showed that a low antioxidant activity is correlated with severe DNA damages in seminal plasma of infertile men [49]. We also noticed that the detrimental effect of oxidative stress on the testicular function was associated with a significant decrease in sex hormones (testosterone, LH and FSH) with an abnormal spermatozoa production. Moreover, the decrease in testicular protein levels could be due to an overproduction of ROS caused by oxidative stress, as reported by Muhammetnur et al. [18]. However, *R. apetalus* decreased lipid peroxidation, but increased SOD and catalase activities, which further confirms its antioxidant property previously reported [27, 28]. These results corroborate those of Raghavendra et al. who demonstrated the in vitro and in vivo antioxidant potentials of *R. apetalus* in diabetic rats [23, 28]. In parallel, *Moringa oleifera* possesses a powerful antioxidant potential in cryptorchid rats by improving the antioxidant enzymes [17, 18]. The antioxidant potential of *R. apetalus* could be justified by the presence of flavonoids, alkaloids and phenols. These phytocomponents are able to stimulate the production of antioxidant enzymes and directly scavenge the ROS [50].

Under cryptorchidism condition, oxidative stress impairs Leydig cell function and causes low testosterone production, leading to abnormal spermatogenesis and infertility [51]. Low LH and FSH also impairs steroidogenesis and spermatogenesis, respectively [52]. The decline in testosterone, LH and FSH levels observed in cryptorchid rats was correlated with abnormal spermatozoa characteristics and therefore indicated an alteration of steroidogenesis and spermatogenesis. Normalization of testosterone level and spermatozoa characteristics in cryptorchid rats given *R. apetalus* could therefore be due to the capacity of the extracts to increase LH and FSH levels. This improvement in sex hormones by *R. apetalus* could again be due to the presence of various phytocomponents such as alkaloids and saponins. These compounds are capable to boost testosterone production [53, 54].

As reported by Muhammetnur et al. [18], we found in the current study that cryptorchidism significantly decreased the diameter of the seminiferous tubes, germ cell thickness and increased peri-vascular fibrosis in the right testis, but did not affect the left testis. These structural damages were positively correlated with oxidative stress, low sex hormone production and high morphological spermatozoa abnormalities. These parameters were improved by *R. apetalus.* Since *R. apetalus* improved structural, reproductive and metabolic parameters in cryptorchid rats; it was of great importance to investigate its effect on fertility. We observed that cryptorchidism decreased the gestation index (62.5%) and fertility index (50%), which could justify the spermatic, hormonal and structural alterations observed in cryptorchid rats. These drops in fertility parameters were reversed by *R. apetalus* extracts. Because cryptorchidism is a congenital anomaly, examination of the effects of *R. apetalus* in juvenile rats is highly needed.

## Conclusion

Taking together, these findings demonstrated that *R. apetalus* boosts fertility by improving spermatozoa characteristics, sex hormone levels, testicular proteins and antioxidant enzymes in cryptorchid rats. The aqueous and methanolic extracts of *R. apetalus* could be considered as promising alternative agents for the management of cryptorchidism related hypofertility. However, a test to verify its safety is required.

## Data Availability

Data are available upon request.

## Abbreviations

CAT: Catalase
CPT: Cryptorchidism
DW: Distilled water
FSH: Follicle stimulating hormone
LH: Luteinizing hormone
MDA: Malondialdehyde
SOD: Superoxide dismutase
